# The Mediated Role of Credibility on Information Sources and Patient Awareness toward Patient Rights

**DOI:** 10.3390/ijerph18168628

**Published:** 2021-08-15

**Authors:** Osnat Roth-Cohen, Shalom Levy, Avi Zigdon

**Affiliations:** 1 School of Communication, Ariel University, Science Park, POB 3, Ariel 40700, Israel; 2 Department of Economics and Business Administration, Ariel University, Science Park, POB 3, Ariel 40700, Israel; shalom@ariel.ac.il; 3 Department of Health Systems Management, School of Health Systems, Ariel University, Science Park, POB 3, Ariel 40700, Israel; aviz@ariel.ac.il

**Keywords:** patient rights, awareness, mass media, public health service, health information interoperability, Israel

## Abstract

Although patient rights are an important issue, this remains an understudied research area. Patients are unaware of their rights, lacking control of health care treatments they might deserve. This can contribute to sustaining inequality as well as failure in achieving welfare policy goals. Drawing on channel complementarity theory, the current study explored patients’ awareness toward their rights, and the credibility of information sources related to patient rights. In a web-based survey, 994 Israeli participants, suffering from chronic illness and using health services, were recruited. To examine the study’s theoretical framework and relationships among the constructs and test the hypotheses, a path analysis was conducted using Structural Equation Modeling. The research model depicts direct and indirect relationships between constructs, and the relevant coefficients. The results show a direct and positive interaction between information credibility and patient rights awareness (β = 0.10, *p* = 0.019). Information credibility partially mediates the relationship between public service information sources and patient rights awareness (bootstrap with 95% CI: 0.01–0.07; *p* = 0.015). The mass media information sources construct is directly and positively related to information credibility (β = 0.36, *p* = 0.000). Age was found as a moderator, indicating that information credibility is a factor only at lower ages. Therefore, patient rights should be systematically and reliably accessible in order to raise the awareness and trust of chronic patients regarding information about patient rights. Using planned health communication campaigns mainly via public service sources that are perceived as trustworthy can help contribute to approach patients more effectively and provide them with accessible and detailed information about their rights.

## 1. Introduction

The media plays an important role in providing accessibility to patient rights [1], which are an inseparable part of human rights. Patient rights promote long-term patient autonomy [2], patient empowerment, the development of health system reforms, and increased awareness for patient participation and inclusion in treatment processes [3]. The principles for advancing patient rights established in 1964 [4] led to a progression in the field of accessibility and realization of patient rights in western societies—both inseparable components of the “right to health” [2]. Failure to provide patient rights information accessibility can disempower patients’ control over their health care treatments as well as cause social gaps and act as a stumbling block for relevant government institutions.

One of the main channels to inform patients about their rights is mass media (e.g., television, radio, newspaper, internet). Although mass media sources convey patient rights information effectively [5], there are those who prefer to receive this information from formal sources such as medical staff (e.g., nurses, doctors, or social workers) or informal sources such as family and patient organizations [6,7,8,9]. As per a European Commission report, a large portion of Europeans will seek to obtain their medical rights from their health insurer or national health service (44%). In addition, 40% will ask their general practitioner or another doctor or specialist and 34% will check the internet (information websites, blogs, or social media) [10]. Yet, chronically ill patients tend to not trust their doctor or the medical staff, and trust similar patients who deliver non-evidence based information about their medical rights [6]. Sources with high credibility have a positive impact on consumer attitudes and behavior [11]. When consumers perceive a source to be credible, they are more likely to accept ideas, resulting in more positive mindsets and behavior attitudes [12,13]. The credibility of health information is also likely to determine the choice of health information sources, which, in turn, may influence an individual’s decisions and behaviors based upon that information [14]. Source credibility, therefore, can act as an effective variable in order to influence consumers.

In light of the need of patients to be aware of their rights, health institutes must learn about information-seeking behavior and possible information channels and how to convey patient rights effectively. Furthermore, research on patient rights is relatively scant [15,16]. The juxtaposition of patients’ awareness of their rights with credibility as a main construct and limited health communication research accentuates the importance of the current study.

This research contributes to understanding the importance and relevance of patient rights awareness, allowing patients accessibility to information that can guide them in the health care system. Credible information sources on patient rights can facilitate relevant and valuable information exchange, and help patients manage uncertainties.

## 2. Patient Rights

Health and healthcare are a fundamental human right [17,18]. Therefore, patient rights are an integral part of the basic right to health and can be defined as the care, treatments, support, and services that the patient needs from the medical, social, and regulatory environment in the process of treatment. In Israel, government and public organizations do not provide accessibility to patient rights information for patients or their family members in a complete and methodical manner. This is especially the case for patients with chronic illness, who do not receive systematic and complete information about their rights [19]. In Israel, studies found that chronic patients expect to receive information about their rights from patient organizations relating to three key aspects: patient rights information, assistance in utilizing patient rights, and financial support for treatments [6]. In the last few years, companies, NGOs, private organizations, and lawyers have begun increasing the accessibility of patient rights information online. The motives for this are diverse: from commercial goals to community contribution following a personal or familial event with the goal of helping the typical citizen learn about their rights. Government organizations are also using this tool to inform the general population about their rights, while continuing their routine activity of providing accessibility to patient rights information. Providing accessibility and utilization of rights is thus a critical issue—half of the eligible population in western European countries do not receive their rights [10]. This causes inequality as well as failure in achieving welfare policy goals, and not using appropriate funds harms progress in this field. Ultimately, providing accessibility to rights by government institutions will assist those who are eligible to utilize their rights, resulting in decreased future expenditure on health services [20].

## 3. Channel Complementarity Theory and Health Information Seeking

Channel complementarity theory suggests that the arrival of a new medium complements the existing channels of information seeking because of underlying interest [21,22,23,24,25]. This idea is different than earlier literature that suggested that the rise of a new medium such as the Internet would displace existing media channels of information seeking [26].

Recent studies used channel complementarity theory to examine health information seeking patterns [23,25,27]. However, only scant studies have applied the theory to examine health information seeking patterns in different countries that differ from the United States, such as Puerto Rico [28] and India [29,30]. Similarly, using channel complementarity theory as a theoretical lens can contribute to the understudied field of patient rights in Israel, as Israel is a unique collectivistic oriented society [31] that is geographically located far from Puerto Rico and India, in the Middle East, and is characterized by a deeply rooted social welfare system [32].

Drawing on channel complementarity theory to investigate health information sources will help us better understand the process through which patients gain information. Ruppel and Rains [23,27] identified several characteristics of health information sources that they argued might function as a basis for source complementarity. They identified four complementarity characteristics: (a) access to medical expertise; (b) tailorability; (c) anonymity; and (d) convenience. In the current research, we present credibility as an important construct when seeking information about patient rights.

## 4. Credibility of Health Information Sources

The credibility of health information is likely to determine the choice of health information sources, which, in turn, may influence patient decisions and behaviors based upon that information [11]. Earlier literature discussed the main role of credibility in the health information domain [13,14,33], illustrating its effect on evaluating health information. Hence, health communication campaigns are likely to increase their effectiveness when they identify and take advantage of information sources that are not only widely accessed by the population, but those that are also recognized as trustworthy channels of communication. Compared to mass media, interpersonal communication is believed to enhance the credibility of health information issues and motivate people to act accordingly to reduce health risk [34]. When consumers perceive a source to be credible, i.e., to possess high levels of expertise and trustworthiness, they are more likely to accept message content, resulting in more positive mindsets and behavior attitudes [12].

## 5. Age and Health Information Seeking

Age has many effects on the patterns of medical information searching, and on the choice of information sources. It even influences medical teams’ patterns of evidence-based medical information use [35,36,37,38,39,40,41]. Studies performed on elderly populations found that older age is significantly correlated to lower health literacy, which in turn impacts search patterns [36,37] and the choice of medical information source [35]. Moreover, elderly people do not utilize credible information supplied by physicians [40], while having a higher likelihood of suffering from chronic illnesses, which leads to a higher dependency on medical information [39]. Combining that with a disadvantage in online health information searching [39,40,41,42] leads to the emergence that age-related processes influence decision-making [29,43]. For example, older adults tend to sample less information and use fewer complex strategies than younger adults [13,44,45]. In addition, older adults appear to adapt to real or perceived declines in cognitive resources by becoming increasingly selective about where they expend cognitive effort. According to earlier research [46,47], older adults’ life experiences, such as social interactions and health decisions, appear to allow them to develop expertise in these areas that may benefit judgment and decision-making. To our knowledge, few health information seeking studies have examined the role of age as a moderating variable [29]; therefore, exploring how age moderates the relationship between patient rights information credibility and patient rights awareness while seeking health information is another contribution of this research.

## 6. Study Framework and Hypotheses

Following the channel complementarity theory [21,22], the current study suggests positive relationships between information sources (e.g., mass media and public health) and patients’ awareness of their rights. However, the study further asserts that information source perceived trustworthiness serves as a significant factor for its use during patient information seeking. Therefore, this study postulates that patients’ need for credible information relates to patients’ awareness of their rights and mediates the relationships between information sources and patients’ awareness of their rights. Finally, given that medical decision-making may differ by patient age, this study posits that age moderates the relationship between patients’ need for credible information and patients’ awareness of their rights. These relationships are presented in the following hypotheses:

**Hypothesis** **H1.** *Mass media information sources have a positive relationship with patient rights awareness*.

**Hypothesis** **H2.** *Public service information sources have a positive relationship with patient rights awareness*.

**Hypothesis** **H3.** *Patient rights information credibility has a positive relationship with patient rights awareness*.

**Hypothesis** **H4.** *Patient rights information credibility mediates the relationship between mass media sources and patient rights awareness*.

**Hypothesis** **H5.** *Patient rights information credibility mediates the relationship between public service information sources and patient rights awareness*.

**Hypothesis** **H6.** *Age moderates the relationship between patient rights information credibility and patient rights awareness. That is, the relationship will be stronger for people of lower age*.

**Figure 1:**
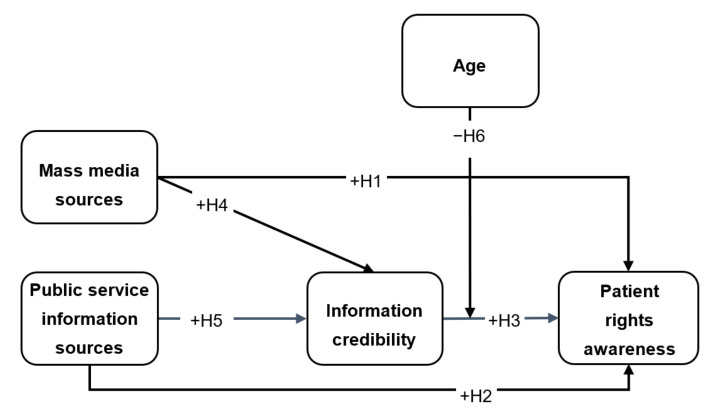
The current study’s theoretical framework, construct relationships, and hypotheses. Mass media information sources and public service information sources are antecedents of patient rights awareness. Information credibility is a mediator and age is a moderator.

Figure 1 illustrates the current study’s theoretical framework, construct relationships, and hypotheses.

## 7. Method

### 7.1. Study Design and Data Collection

Data were collected through a national web-based survey. Invitations to participate were distributed to the general population over the age of 18 by research assistants through social media platforms, including an individual disposable non-reusable link to the questionnaire. Written informed consent was obtained from all participants prior to the questionnaire. Ariel University’s research ethics committee (ref AU-HEA-AZ-20170730) reviewed and approved all aspects of this study. The self-reported questionnaires were coded for anonymous data analysis.

A preliminary requirement for participating in this study was having a chronic illness and using health services. Out of 8839 respondents who entered the survey’s website, 994 complied with research requirements and formed the sample of the study.

The sample size estimation was based upon data of the general population and chronically ill patients (21%) from the Israel Central Bureau of Statistics (CBS). Assuming that the incidence of chronic diseases is estimated at about 21% of the general population in Israel, and the population of Israel at the end of 2020 is estimated at about 9,291,000 [48], the population of chronically ill patients in Israel is estimated at 1,951,111. At a confidence level of 95%, when the rate in the population is unpredictable and taken as 50% (maximum), at a significance level of 5%, a sample size for the purpose of this study was calculated based upon these variables to be approximately 384 participants. The sample used in this study highly exceeds the satisfying sample size.

### 7.2. Measures

For the dependent variable, *patient rights awareness*, a set of 24 questions was designed to capture varied patient rights that belong to three different categories: (1) social security rights (e.g., eligibility for a nursing benefit, income support, mobility allowance, etc.); (2) health care system rights (e.g., eligibility for benefits for patients with various diseases, ceiling payment for medications for chronic patients, medicines based on the health basket, etc.); and (3) other rights (e.g., eligibility to employ a foreign nursing worker, for income tax exemption for those with a high medical disability, for a discount on public transportation, etc.). Respondents were asked to indicate their familiarity with the different patient rights on a six-point Likert scale, ranging from 1 = very unfamiliar to 6 = very familiar. Independent variables were adopted from Slaughter and Turban [49] and Remmerswaal and Muris [50]. The *information credibility* scale was based on three items that express perception of the credibility of patient rights general information: (1) a publication detailing information, data, and facts on patient rights will include reliable information; (2) a published article (i.e., editorial) on patient rights is more credible than an advertisement because it is not sponsored by an interested party; and (3) an advertisement for patient rights funded by an institutional body will provide reliable information. The *mass media* *information sources* scale was based on three items expressing mass media preference as a source for patient rights information: (1) I prefer to receive information about my patient rights in advertisements on various mass media channels (e.g., TV, radio, newspaper, Internet); (2) I prefer to receive information about my patient rights in articles/programs on various mass media channels (e.g., TV, radio, newspaper, Internet); and (3) I prefer to receive information about my patient rights on social media. The *public service information sources* scale was also based on three items expressing preference for health authority databases and services as sources for medial rights information: (1) I prefer to receive information about my patient rights on the website of the social security service/health services; (2) I prefer to receive the information about my patient rights directly from the clerk at my health services branch; and (3) I prefer to receive information about my patient rights from a health service/social security information booklet. The respondents were asked to indicate their level of agreement with different statements. A six-point Likert scale was used, ranging from 1 = strongly disagree to 6 = strongly agree. Demographic and health related data were also collected.

## 8. Results

The sample was 71 percent female, aged 18–96 years, with an average age of 41. Most were married or lived with a partner (60%). The education level of the majority of the participants was above high school (63%), with 53% reporting a below-average income (Table 1).

### 8.1. Validity and Reliability

Given the exploratory study, the sample was split in order to estimate construct validity and reliability. First, in half of the sample, items were subjected to exploratory factor analysis (EFA) via SPSS. Six factors were produced, explaining 59.55 percent of the cumulative variance, and almost all item loadings were above 0.5, with one above 0.4, which is acceptable in an exploratory study [51]. Next, in the other half, the six variable items were subjected to confirmatory factor analysis (CFA), using AMOS-25 Program, for estimating construct validity and reliability. The results confirm the constructs for the overall CFA (χ2 value (438) = 861.31, *p* = 0.000 (χ2/df < 2); Comparative Fit Index (CFI) = 0.952; Normed Fit Index (NFI) = 0.907; and Root Mean Square Error of Approximation (RMSEA) = 0.044). The CFA shows that scale items loaded satisfactorily on the relevant latent variables. The standardized regression estimates of the six constructs were above 0.50, reflecting acceptable fit of the measures.

Next, the dependent variable, patient rights awareness, is a construct of three facets. A second confirmatory factor analysis (CFA) was conducted on the three facets of patient rights awareness to estimate the second-order construct validity. The results confirm the second-order construct (χ2 value (209) = 448.04, *p* =000 0.05 (χ2/df < 3); Comparative Fit Index (CFI) = 0.968; Normed Fit Index (NFI) = 0.943; and Root Mean Square Error of Approximation (RMSEA) = 0.048). The CFA shows that the constructs loaded satisfactorily: the loadings were 0.82, 0.96, and 0.99 (above 0.5). Therefore, the second-order patient rights awareness construct is significantly fitted for further model testing.

Finally, CFA was measured while the dependent variable, patient rights awareness, was treated as a second-order construct. Here, the results also confirm the constructs (χ2 value (438) = 860.44, *p* = 0.000 (χ2/df < 2); Comparative Fit Index (CFI) = 0.952; Normed Fit Index (NFI) = 0.908; and Root Mean Square Error of Approximation (RMSEA) = 0.044) and the standardized regression estimates were above 0.50. For this CFA measure, Average Variance Extracted (AVE) and Composite Reliability (CR) were calculated (see Table 2). An acceptable level of convergent validity (AVE) was found for most of the measurements, except for two measurement that were close to the acceptable level of 0.5. Internal consistency (CR above 0.6) was found in all measurements. Additionally, composite reliability measures are higher than 0.6; therefore, the convergent validity of the constructs is satisfactory [35,52]. Comparing the square of the correlation estimate between any pair of these study constructs with the Average Variance Extracted (AVE) values reveals greater values for AVE in all cases, which verifies the discriminant validity of the constructs (Table 3 shows the correlation patterns between variables and the Maximum Shared squared Variance (MSV)).

The internal consistency was further examined using Cronbach’s alpha. All constructs of patient rights awareness (social security rights, health care system rights, and other rights) achieved high reliability of measurement (Cronbach’s alphas of 0.90, 0.90, and 0.87, respectively). The independent variables of information credibility, mass media information sources, and public service information sources displayed acceptable reliability of measurement (Cronbach’s alphas of 0.68, 0.80, and 0.62, respectively), with Cronbach’s alpha above 0.6 being acceptable in an exploratory study [51].

### 8.2. Model Testing

To examine the relationships among the constructs (Table 4) and test the hypotheses, a path analysis was conducted using Structural Equation Modeling (SEM) using AMOS-25 Program. In order to create interaction variables for the age moderation check, a three-step procedure [53] was followed to standardize the relevant independent variables. The procedure suggested by Bagozzi and Edwards was followed, and a number of alternative models were compared [54]. The model that gave a better fit was retained as the final model.

**Figure 2:**
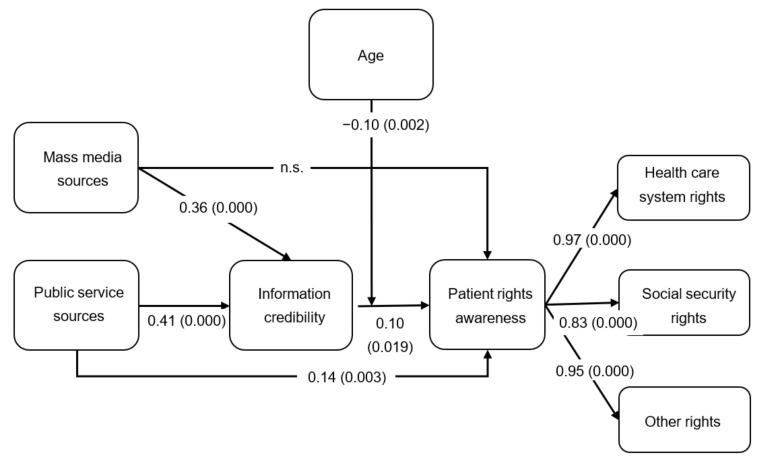
Awareness of patient rights: a path model ^a^. Mass media information sources and public service information sources are antecedents of patient rights awareness. Information credibility is a mediator and age is a moderator. ^a^ Path parameters are standardized parameter estimates and only significant paths are shown. Significance is displayed in parenthesis.

The overall fit statistics (goodness of fit measures) exhibit an acceptable level of fit (*χ*^2^ value (489) = 1431.55, *p* = 0.000 (*χ*2/*df* < 3); Comparative Fit Index (CFI) = 0.944; Normed Fit Index (NFI) = 0.917; Root Mean Square Error of Approximation (RMSEA) = 0.044), indicating that the path model is valid. The path model, regression standardized coefficients, and their significance are illustrated in Figure 2. Table 2 summarizes the relationships between the model’s constructs.

The model depicts direct and indirect relationships between constructs, and the relevant coefficients. As indicated by Figure 2, information credibility is directly and positively related to patient rights awareness (β = 0.10, *p* = 0.019). Therefore, Hypothesis H3 is supported.

The mass media information sources construct is directly and positively related to information credibility (β = 0.36, *p* = 0.000); however, no direct relationship was found with patient rights awareness. Additionally, the relationship between mass media information source and patient rights awareness was actually indirect (β(c-c′) = 0.04) and fully mediated by credibility (bootstrap with 95% CI: 0.01–0.07; *p* = 0.015; Sobel test Z = 2.19, *p* = 0.028). Table 5 summarizes the mediation analysis measures. Thus, Hypothesis H4 was supported while Hypothesis H1 was not supported.

The public service information sources construct displays both direct (β = 0.14, *p* = 0.003) and indirect (β(c-c′) = 0.04) relationships with patient rights awareness (displaying a total relationship of β = 0.18), and also a direct relationship with credibility (β = 0.41, *p* = 0.000). That is, credibility partially mediates the relationship between public service information sources and patient rights awareness (bootstrap with 95% CI: 0.01–0.07; *p* = 0.017; Sobel test Z = 2.23, *p* = 0.026). Hence, Hypotheses H2 and H5 were supported.

**Figure 3:**
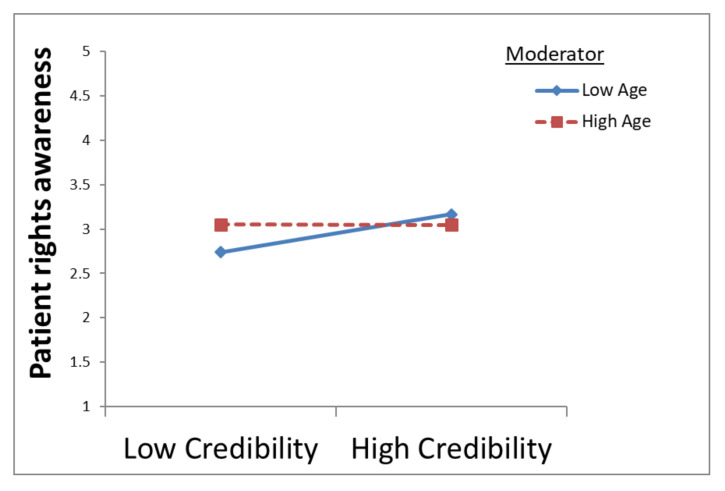
The moderation effect of age.

The regression results also show a moderation effect of age. The age and information credibility interaction variable has a negative relationship with patient rights awareness (β = −0.10, *p* = 0.002). This indicates that age dampens the positive relationship between credibility and patient rights awareness, which means that credibility has a significantly stronger positive effect on patient rights awareness at lower ages (Figure 3). In other words, credibility is a factor only at lower ages, supporting Hypothesis H6.

## 9. Discussion

Patient rights are an integral part of the basic right to human health, empowering patients to make informed decisions about their health services and treatments and contributing to achieving welfare policy goals. The present study provides a novel understanding of patients’ awareness of their rights and the importance of credibility as a mediator of patient rights information sources. Examining the process through which patients select and use multiple sources when acquiring health information can help us better understand the role of sources in health information-seeking behavior [27].

Sources produce information that may be vital to chronic patients when seeking health information through various channels. Patient awareness of sources for health information is necessary for the design of effective health information services and information systems [9]. However, no direct relationship was found between mass media information source and patient rights awareness (H1). This finding is surprising as a lack of patient rights awareness might lead to receiving unsuitable treatment and influence patient access to services, causing social gaps and acting as a stumbling block for relevant government institutions [19]. The issue of a lack of patient rights awareness is of high importance, since health decision-making poses several challenges to patients such as acting under distress, uncertainty, and often under time pressure.

Our findings illustrate the central role of credibility, as we found a direct and positive interaction between information credibility and patient rights awareness (H3). Relying on a credible source for conveying patient rights might create beneficial relationships in which the patients will be more aware of their rights, allowing them to optimize their decision-making process in managing their illness. Moreover, awareness of patient rights is important for showing progress in building positive relations [55] with government and public organizations and enhancing patients’ knowledge in relation to their rights and access to rights-related information. Since mass media channels convey persuasive messages, a possible explanation is that mass media sources lack the attributes of trustworthiness and reliability and are perceived by consumers (i.e., patients) as unreliable. Indeed, some media platforms are viewed with more skepticism [56]. Moreover, low mass media credibility may negatively affect perceptions of organizations due to the persuasive intent and ambiguity of content sources [57]. Consequently, patients do not adopt information directly transferred via mass media but obtain awareness only when mediated with credibility.

Our findings also reveal both direct and indirect relationships with patient rights awareness for public service information sources and a direct relationship with credibility. That is, credibility partially mediates the relationship between public service information sources and patient rights awareness (H2 and H5). This mirrors earlier research findings that information from government and noncommercial entities is viewed as having higher levels of trustworthiness and expertise [14,58] and can be explained by the fact that public health sources (i.e., health care system, social security) are considered authoritative in the field of health care. Identified as health care providers, they are, therefore, initially perceived as credible. Earlier research found that credibility lies foremost in the trustworthiness and expertise of the source itself [59]. This explains why patients may feel supported and empowered by a reliable information source such as a health care provider (e.g., nurses, doctors, or social workers). Moreover, users tend to transfer the reputation of the information source to the content itself [60]. Thus, public services are more associated with patient rights-related objectivity and specialized expertise, reflecting why they are relied on directly as information sources. This strengthens our finding that public service information sources display both direct and indirect relationships with patient rights awareness. Moreover, we may assume that complementary relationships exist between health information seeking from both information sources: mass media and public health (i.e., interpersonal), with patients seeking health information from interpersonal sources also being more likely to seek health information from mass media as compared to patients who did not use these public health sources.

Credibility is perceived as vital as it challenges the individual to evaluate and select trustworthy information sources. As such, the mass media information source is directly and positively related to information credibility (H4). That is, the relationship between mass media information source and patient rights awareness was indirect and fully mediated by credibility. Patients do not rely on mass communication tools and need to assess their credibility and reliability before considering the data. When patients perceive a source as credible (i.e., to possess high levels of expertise and trustworthiness), they are more likely to accept its message content, resulting in more positive mindsets and attitudes [61]. Only then can the usefulness of the information take root and ultimately lead to higher awareness of patient rights.

Our study indicates that credibility has a significantly stronger positive effect on patient rights awareness in low age groups (H6—Figure 3). This is interesting since chronic disease is associated with older age [9], and it can be assumed that this would influence high age source credibility evaluation. According to earlier research [46,47], older adults’ life experiences, such as social interactions and health decisions, appear to allow them to develop expertise in areas that may benefit judgment and decision-making. When older adults are faced with decisions or judgments in health contexts they frequently encounter, their previous experience may enable them to avoid bias that affects younger adults in the same decisions [62]. In addition, older adults tend to sample less information and use less complex strategies than younger adults and have a more difficult time multitasking than younger ones [63], thus showing that high age group awareness of patient rights will rely on less information seeking, without considering its credibility.

This research makes several contributions. First, it provides a theoretical contribution by suggesting credibility as a complementarity characteristic of sources, which is argued to serve as a basis for source use during information seeking [56]. Earlier research [23,27] presented four characteristics: access to medical expertise, tailorability, anonymity, and convenience, disregarding the trustworthiness construct. Second, to the best of our knowledge, patients’ awareness toward their rights and the credibility of information sources has yet to be adequately studied. This is surprising, as in order to be aware of patient rights, the reliability of the information source is necessary in addition to the information itself. Credibility is, therefore, an essential feature as it facilitates the exchange and use of information due to the increased perceived reliability when the information source is trusted [34]. Third, this is a timely study discussing the effects of credibility on patient awareness toward patient rights. As we are currently facing an ongoing global health crisis, the issue of patient rights is becoming more crucial than ever. People are seeking information in relation to their health rights (e.g., the COVID-19 vaccine; at risk population restrictions). Health institutions looking to reach constituents during the COVID-19 pandemic or future crises can use this study’s findings for better planning of effective health communication campaigns. For example, a planned health communication campaign that is conveyed using public service sources perceived as trustworthy can help contribute to approaching patients more effectively and providing them with accessible and detailed information about their rights.

Although adding to the limited research on patient rights awareness and source credibility, this study’s main limitation includes the fact that patient illness or health status was self-reported, and their medical files were not checked. Further research should compare participants with chronic illness that use health services with healthy people that do not confront specific illness in order to learn more about their perceptions toward patient rights awareness and information source credibility. Additionally, we tested participant attitudes toward mass media as a whole, and so future research that distinguishes media channel types (off-line vs. on-line) is warranted. When discussing optional information sources, additional future research should investigate the role of interpersonal communication—whether it enhances the credibility of information on health issues and motivates people to act accordingly.

## 10. Conclusions

This research discussed the understudied topic of patient rights awareness and revealed the relationships between patient rights awareness and information credibility. Despite earlier studies that found awareness to be an important factor in the decision-making process, this research highlights that evaluating information as credible is highly significant since it affects health-related issues. Based on channel complementarity theory, we observed complementary patterns with mass media as well as public health channels. Complementarity is important as participants change sources during a search, thus illustrating Lin and Dutta’s notion that “the complementarity lens posits that different channels can be integrated into a broader media ecosystem, performing complementary functions to each other”.

Using channel complementarity theory [27,56], this study achieved several objectives: First, to better understand what influences patients’ awareness toward their rights, we presented a conceptual model that will assist health institutions with providing patient rights via information sources. Second, the earlier literature neglected patient rights as a research area. This is surprising as unawareness of patient rights may disempower patients’ control over their health care treatments, aggravate inequality, and undermine the poverty-reducing capacity of the welfare state. This research aim was to bridge this gap and add to patient rights literature, by focusing on credibility as a main construct when choosing information sources related to patient rights.

Future research can further use Rains and Ruppel [27] channel complementarity theory to investigate the relationships between the four complementarity characteristics (i.e., access to medical expertise, tailorability, anonymity, and convenience) and information credibility in order to learn more about the role of credibility in patient rights.

### Practice Implications

The credibility of health information may influence patient decisions and behaviors based on the information source. Hence, in order to inform patients of their rights, health communication campaigns are likely to increase their effectiveness when they identify and take advantage of information sources that are recognized as direct trustworthy channels of communication. We advise government and public organizations to use public health sources as they possess direct credibility and are not perceived as commercial mass communication channels.

Since fewer barriers to source credibility exist for the elderly, when approaching older people on patient rights-related issues, information sources focusing on emotional content should perhaps be privileged as this population tends to sample less information and uses fewer complex strategies than younger adults. Since older adults appear to be more selective about where they expend cognitive effort, health care providers should adopt this patient rights transmission approach while diminishing strictly rational information.

## Figures and Tables

**Table 1:** Respondent characteristics (*n* = 994).

Respondent Characteristics	*n* (%)
Gender	
Female	711 (71.5)
Male	283 (28.5)
Age (years)	
18–24	211 (21.2)
25–34	190 (19.1)
35–44	166 (16.7)
45–54	210 (21.1)
55–64	143 (14.4)
65+	74 (7.5)
Marital Status	
Married/Partner	596 (60.0)
Bachelor/Never Married	275 (27.7)
Divorced	89 (9.0)
Widowed	28 (2.8)
Others	6 (0.6)
Country of Birth	
Israel	657 (66.1)
Other	337 (33.9)
Education Background	
Primary education	51 (5.1)
Secondary education	292 (29.3)
Further education	131 (13.2)
University degree and higher	499 (50.2)
Other	22 (2.2)
Income	
Considerably below average	298 (30.0)
Slightly below average	230 (23.1)
Average (10,000 Shekels)	184 (18.5)
Slightly above average	202 (20.3)
Considerably above average	61 (6.2)
Would rather not say	19 (1.9)
Reported Health Status	
Very good	183 (18.4)
Good	519 (52.2)
Not so good	229 (23.0)
Not good	48 (4.9)
Bad	15 (1.5)

**Table 2:** CFA—items’ factor loading range, variables’ reliability and validity measures.

	Std. Coef.	AVE	CR
Patient rights awareness—general	0.82 *–0.99 *	0.86	0.95
Social security rights	0.72 *–0.82 *	0.58	0.91
Health care system rights	0.60 *–0.75 *	0.46	0.88
Other rights	0.67 *–0.75 *	0.50	0.89
Information credibility	0.54 *–0.80 *	0.50	0.74
Mass media information sources	0.56 *–0.84 *	0.57	0.79
Public service information sources	0.60 *–0.73 *	0.42	0.68

* Standardized Coefficients, *p* < 0.01; AVE = Average Variance Extracted; CR = Composite Reliability.

**Table 3:** Correlations ^a^ between variables and the Maximum Shared squared Variance (MSV).

		1	2	3	4
1	Patient rights awareness	0.86	0.126 **	0.084 **	0.129 **
2	Information credibility	0.016	*0.50*	0.357 **	0.276 **
3	Mass media information sources	0.001	0.127	0.57	0.181 **
4	Public service information sources	0.017	0.076	0.033	*0.42*

** *p* < 0.01; ^a^ Correlations are in the upper right side while the MSV are in the lower left side. AVE (Average Variance Extracted) are in the diagonal italics.

**Table 4:** Relationships between the model’s constructs: direct and indirect.

Relationships	Standardized Effect (β)			Regression Weights (Direct)		
	Total	Direct	Indirect	Estimate (B)	C.R. (t)	*p*
Public service sources → Patient rights awareness	0.183	0.142	0.041 *	0.258	2.977	0.003
Mass media sources → Patient rights awareness	0.036	0.000	0.036 **			
Information credibility → Patient rights awareness	0.100	0.100	0.000	0.105	2.336	0.019
Mass media sources → Information credibility	0.359	0.359	0.000	0.313	9.035	0.000
Public service sources → Information credibility	0.405	0.405	0.000	0.699	7.890	0.000
Age × Information credibility → Patient rights awareness	−0.101	−0.101	0.000	−0.109	−3.059	0.002

* *p* = 0.017, * *p* = 0.015, for indirect effects.

**Table 5:** Mediation analysis.

Path	Std. Coef. (β)	S.E.	Bootstrap with 95% CI
**A**			
Mass media information sources → Information credibility (a)	0.359	0.043	0.292–0.428 **
Information credibility → Patient rights awareness (b)	0.100	0.044	0.034–0172 *
Total effect: Mass media information sources → Patient rights awareness (c)	0.036	0.017	0.012–0.070 *
Direct effect: Mass media information sources → Patient rights awareness (c′)	0.000	0.000	--
**B**			
Public service information sources → Information credibility (a)	0.405	0.037	0.348–0.474 **
Information credibility → Patient rights awareness (b)	0.100	0.044	0.034–0.172 *
Total effect: Public service information sources → Patient rights awareness (c)	0.183	0.043	0.104–0.252 *
Direct effect: Public service information sources → Patient rights awareness (c′)	0.142	0.050	0.061–0.227 *

Notes: * *p* < 0.05, ** *p* < 0.01; CI = Confidence Interval.

## References

[1] Eurofound (2015). Access to Social Benefits: Reducing Non-Take-Up.

[2] World Health Organization (2010). Patient Safety and Rights. Developing Tools to Support Consumer Health Literacy. www.euro.who.int/__data/assets/pdf_file/0018/133128/e94739.pdf.

[3] Palm W., Townend D., Nys H. (2016). Patients’ Rights in the European Union: From Recognition to Implementation. Eur. J. Public Health.

[4] World Medical Association (2013). World Medical Association Declaration of Helsinki: Ethical Principles for Medical Research Involving Human Subjects: Ethical Principles for Medical Research Involving Human Subjects. JAMA.

[5] Lee S.T., Lin J. (2020). The Influence of Offline and Online Intrinsic Motivations on Online Health Information Seeking. Health Commun..

[6] Zigdon A., Eckhaus E., Lerer R., Rosenfeld M. Patients’ Perspectives on Services and Activities of Patient Organizations–Development of a Patient-Oriented Questionnaire. Chronic Illn.

[7] Unnikrishnan B., Trivedi D., Kanchan T., Rekha T., Mithra P., Kumar N., Kulkarni V., Holla R., Talish M. (2017). Patients’ Awareness about Their Rights: A Study from Coastal South India. Sci. Eng. Ethics.

[8] Getz I., Weissman G. (2010). An Information Needs Profile of Israeli Older Adults, Regarding the Law and Services. J. Libr. Inf. Sci..

[9] Zhang Y. (2014). Beyond Quality and Accessibility: Source Selection in Consumer Health Information Searching: Beyond Quality and Accessibility: Source Selections in Consumer Health Information Searching. J. Assoc. Inf. Sci. Technol..

[10] Eurobarometer (2015). Special Eurobarometer 425: Patients’ Rights in Cross-Border Healthcare in the European Union 2015. https://data.europa.eu/data/datasets/s2034_82_2_425_eng?locale=en.

[11] Mishra A.S., Roy S., Bailey A.A. (2015). Exploring Brand Personality-Celebrity Endorser Personality Congruence in Celebrity Endorsements in the Indian Context: Brand-Celebrity Personality. Psychol. Mark..

[12] Biswas D., Biswas A., Das N. (2006). The Differential Effects of Celebrity and Expert Endorsements on Consumer Risk Perceptions. The Role of Consumer Knowledge, Perceived Congruency, and Product Technology Orientation. J. Advert..

[13] Choi W. (2020). Older Adults’ Credibility Assessment of Online Health Information: An Exploratory Study Using an Extended Typology of Web Credibility. J. Assoc. Inf. Sci. Technol..

[14] Sun Y., Zhang Y., Gwizdka J., Trace C.B. (2019). Consumer Evaluation of the Quality of Online Health Information: Systematic Literature Review of Relevant Criteria and Indicators. J. Med. Internet Res..

[15] Putturaj M., Van Belle S., Criel B., Engel N., Krumeich A., Nagendrappa B.P., Prashanth N.S. (2020). Towards a Multilevel Governance Framework on the Implementation of Patient Rights in Health Facilities: A Protocol for a Systematic Scoping Review. BMJ Open.

[16] Abedi G., Shojaee J., Moosazadeh M., Rostami F., Nadi A., Abedini E., Palenik C.J., Askarian M. (2017). Awareness and Observance of Patient Rights from the Perspective of Iranian Patients: A Systematic Review and Meta-Analysis. Iran. J. Med. Sci..

[17] Hobdell M.H. (1996). Health as a Fundamental Human Right. Br. Dent. J..

[18] Epstein-Lubow G. (2020). Care Is A Basic Human Right: A Review of Arthur Kleinman’s ‘The Soul of Care’, a Compelling Depiction of Family Caregiving with Important Implications for People Living with Dementia and Everyone Who Supports Them. Health Aff..

[19] State Comptroller (2015). Annual Report (65C) and Accounts for the Fiscal Year 2013, Non-Take-up of Social Rights. 65C. www.mevaker.gov.il/he/Reports/Report_290/ReportFiles/fullreport_2.pdf.

[20] Janssens J., Van Mechelen N. (2017). Who Is to Blame? An Overview of the Factors Contributing to the Non-Take-up of Social Rights.

[21] Dutta-Bergman M.J. (2004). Complementarity in Consumption of News Types across Traditional and New Media. J. Broadcast. Electron. Media.

[22] Dutta-Bergman M.J. (2004). Interpersonal Communication after 9/11 via Telephone and Internet: A Theory of Channel Complementarity. New Media Soc..

[23] Ruppel E.K., Rains S.A. (2012). Information Sources and the Health Information-Seeking Process: An Application and Extension of Channel Complementarity Theory. Commun. Monogr..

[24] Lai C.-H. (2014). An Integrated Approach to Untangling Mediated Connectedness with Online and Mobile Media. Comput. Human Behav..

[25] Ruppel E.K., Burke T.J. (2015). Complementary Channel Use and the Role of Social Competence. J. Comput. Mediat. Commun..

[26] Dimmick J., Chen Y., Li Z. (2004). Competition between the Internet and Traditional News Media: The Gratification-Opportunities Niche Dimension. J. Media Econ..

[27] Rains S.A., Ruppel E.K. (2016). Channel Complementarity Theory and the Health Information-Seeking Process: Further Investigating the Implications of Source Characteristic Complementarity. Communic. Res..

[28] Tian Y., Robinson J.D. (2014). Media Complementarity and Health Information Seeking in Puerto Rico. J. Health Commun..

[29] Lin J., Dutta M.J. (2017). A Replication of Channel Complementarity Theory among Internet Users in India. Health Commun..

[30] Neyazi T.A., Kumar A., Dutta M.J. (2019). Channel Complementarity or Displacement? Theory and Evidence from a Non-Western Election Context. J. Broadcast. Electron. Media.

[31] Roth-Cohen O., Tamir I. (2017). ‘The Winner Takes It All’: Values and Benefits of Israeli Sports Gambling Advertisements. Int. J. Hist. Sport.

[32] Gal J., Madhala S. The Social Welfare System and the Coronavirus Crisis: An Overview. www.taubcenter.org.il/wp-content/uploads/2021/01/socialwelfareandthecoronaviruscrisisoverviewheb.pdf.

[33] Chang Y.-S., Zhang Y., Gwizdka J. (2021). The Effects of Information Source and EHealth Literacy on Consumer Health Information Credibility Evaluation Behavior. Comput. Human. Behav..

[34] Mertens F., Távora R., Nakano E.Y., Castilhos Z.C. (2017). Information Sources, Awareness and Preventive Health Behaviors in a Population at Risk of Arsenic Exposure: The Role of Gender and Social Networks. PLoS ONE.

[35] Fornell C., Larcker D.F. (1981). Evaluating Structural Equation Models with Unobservable Variables and Measurement Error. J. Mark. Res..

[36] MacLeod S., Musich S., Gulyas S., Cheng Y., Tkatch R., Cempellin D., Bhattarai G.R., Hawkins K., Yeh C.S. (2017). The Impact of Inadequate Health Literacy on Patient Satisfaction, Healthcare Utilization, and Expenditures among Older Adults. Geriatr. Nurs..

[37] Van Hoa H., Giang H.T., Vu P.T., Van Tuyen D., Khue P.M. (2020). Factors Associated with Health Literacy among the Elderly People in Vietnam. Biomed Res. Int..

[38] Zigdon A., Zigdon T., Moran D.S. (2020). Attitudes of Nurses towards Searching Online for Medical Information for Personal Health Needs: Cross-Sectional Questionnaire Study. J. Med. Internet Res..

[39] Kovner C.T., Mezey M., Harrington C. (2002). Who Cares for Older Adults? Workforce Implications of an Aging Society. Health Aff..

[40] Chen X., Hay J.L., Waters E.A., Kiviniemi M.T., Biddle C., Schofield E., Li Y., Kaphingst K., Orom H. (2018). Health Literacy and Use and Trust in Health Information. J. Health Commun..

[41] Song X., Song S., Chen S., Zhao Y., Zhu Q. (2019). Factors Influencing Proxy Internet Health Information Seeking among the Elderly in Rural China: A Grounded Theory Study. Human Aspects of IT for the Aged Population. Design for the Elderly and Technology Acceptance.

[42] Atkinson N.L., Saperstein S.L., Pleis J. (2009). Using the Internet for Health-Related Activities: Findings from a National Probability Sample. J. Med. Internet Res..

[43] Gehrau V., Fujarski S., Lorenz H., Schieb C., Blöbaum B. (2021). The Impact of Health Information Exposure and Source Credibility on COVID-19 Vaccination Intention in Germany. Int. J. Environ. Res. Public Health.

[44] Robertson-Lang L., Major S., Hemming H. (2011). An Exploration of Search Patterns and Credibility Issues among Older Adults Seeking Online Health Information. Can. J. Aging.

[45] Seo H., Blomberg M., Altschwager D., Vu H.T. (2021). Vulnerable Populations and Misinformation: A Mixed-Methods Approach to Underserved Older Adults’ Online Information Assessment. New Media Soc..

[46] Tentori K., Osherson D., Hasher L., May C. (2001). Wisdom and Aging: Irrational Preferences in College Students but Not Older Adults. Cognition.

[47] Kim S., Hasher L. (2005). The Attraction Effect in Decision Making: Superior Performance by Older Adults. Q. J. Exp. Psychol. A.

[48] Central Bureau of Statistics (CBS) Population of Israel on the Eve of 2021. www.cbs.gov.il/en/mediarelease/Pages/2020/Population-of-Israel-on-the-Eve-of-2021.aspx.

[49] Slaughter J.E., Cable D.M., Turban D.B. (2014). Changing Job Seekers’ Image Perceptions during Recruitment Visits: The Moderating Role of Belief Confidence. J. Appl. Psychol..

[50] Remmerswaal D., Muris P. (2011). Children’s Fear Reactions to the 2009 Swine Flu Pandemic: The Role of Threat Information as Provided by Parents. J. Anxiety Disord..

[51] Hair J.F., Anderson R.E., Babin B.J., Black W.C. (2009). Multivariate Data Analysis: A Global Perspective.

[52] Lam L.W. (2012). Impact of Competitiveness on Salespeople’s Commitment and Performance. J. Bus. Res..

[53] Cortina J.M., Chen G., Dunlap W.P. (2001). Testing Interaction Effects in LISREL: Examination and Illustration of Available Procedures. Organ. Res. Methods.

[54] Bagozzi R.P., Edwards J.R. (1998). A General Approach for Representing Constructs in Organizational Research. Organ. Res. Methods.

[55] Duff B.R.-L., Sar S. (2015). Seeing the Big Picture: Multitasking and Perceptual Processing Influences on Ad Recognition. J. Advert..

[56] Metzger M.J., Flanagin A.J., Medders R.B. (2010). Social and Heuristic Approaches to Credibility Evaluation Online. J. Commun..

[57] Wu M., Huang Y., Li R., Bortree D.S., Yang F., Xiao A., Wang R. (2016). A Tale of Two Sources in Native Advertising: Examining the Effects of Source Credibility and Priming on Content, Organizations, and Media Evaluations. Am. Behav. Sci..

[58] Choi W., Stvilia B. (2015). Web Credibility Assessment: Conceptualization, Operationalization, Variability, and Models. J. Assoc. Inf. Sci. Technol..

[59] Tseng S., Fogg B.J. (1999). Credibility and Computing Technology. Commun. ACM.

[60] Shen C., Kasra M., Pan W., Bassett G.A., Malloch Y., O’Brien J.F. (2019). Fake Images: The Effects of Source, Intermediary, and Digital Media Literacy on Contextual Assessment of Image Credibility Online. New Media Soc..

[61] Toncar M., Reid J.S., Anderson C.E. (2007). Effective Spokespersons in a Public Service Announcement: National Celebrities, Local Celebrities and Victims. J. Commun. Manag..

[62] Peters E., Diefenbach M.A., Hess T.M., Västfjäll D. (2008). Age Differences in Dual Information-Processing Modes: Implications for Cancer Decision Making: Implications for Cancer Decision Making. Cancer.

[63] Brasel S.A., Gips J. (2011). Media Multitasking Behavior: Concurrent Television and Computer Usage. Cyberpsychol. Behav. Soc. Netw..

